# Antibiotic use in children under 5 years of age in Northern Tanzania: a qualitative study exploring the experiences of the caring mothers

**DOI:** 10.1186/s13756-022-01169-w

**Published:** 2022-11-03

**Authors:** Matilda Emgård, Rose Mwangi, Celina Mayo, Ester Mshana, Gertrud Nkini, Rune Andersson, Margret Lepp, Susann Skovbjerg, Florida Muro

**Affiliations:** 1 Department of Infectious Diseases, Institute of Biomedicine, Sahlgrenska Academy, University of Gothenburg, Gothenburg, Sweden; 2 Centre for Antibiotic Resistance Research (CARe), University of Gothenburg, Gothenburg, Sweden; 3 Department of Paediatrics, Queen Silvia Children’s Hospital, Sahlgrenska University Hospital, Gothenburg, Region Västra Götaland Sweden; 4 Institute of Public Health, Kilimanjaro Christian Medical University College (KCMUCo), Sokoine Road, Moshi, Tanzania; 5 Department of Community Health, Kilimanjaro Christian Medical Centre (KCMC), Moshi, Tanzania; 6 Department of Clinical Microbiology, Sahlgrenska University Hospital, Gothenburg, Region Västra Götaland Sweden; 7 Institute of Health and Care Sciences, Sahlgrenska Academy, University of Gothenburg, Gothenburg, Sweden; 8 Østfold University College, Halden, Norway; 9 School of Nursing and Midwifery, Griffith University, Gold Coast, QLD Australia; 10 Faculty of Social and Health Sciences, Inland Norway University of Applied Sciences, Elverum, Norway; 11 Faculty of Medicine, Public Health & Nursing, Universitas Gadjah Mada, Yogyakarta, Indonesia

**Keywords:** Antimicrobial stewardship, Drug resistance, Bacterial, Africa, Eastern, Research, Qualitative, Focus groups

## Abstract

**Background:**

Antimicrobial resistance is a serious threat to the global achievements in child health thus far. Previous studies have found high use of antibiotics in children in Northern Tanzania, but the experiences of the primary care-givers, who play a key role in accessing and administering antibiotics for the sick child, have remained largely unknown. Therefore, the aim of this study was to understand mothers’ conceptions of antibiotic use in their children, which is of importance when forming strategies to improve antibiotic use in the community.

**Method:**

A qualitative study including eight focus group discussions with mothers of under-five children in Moshi urban and rural districts, Northern Tanzania, was performed during 2019. The discussions were recorded, transcribed verbatim, translated into English and analysed according to the phenomenographic approach.

**Findings:**

Three conceptual themes emerged during analysis; (1) conceptions of disease and antibiotics, (2) accessing treatment and (3) administering antibiotics. Antibiotics were often perceived as a universal treatment for common symptoms or diseases in children with few side-effects. Although mothers preferred to attend a healthcare facility, unforeseen costs, long waits and lack of financial support from their husbands, posed barriers for healthcare seeking. However, pharmacies were perceived as a cheap and convenient option to access previously used or prescribed antibiotics. Some mothers sought advice from a trusted neighbour regarding when to seek healthcare, thus resembling the function of the community health worker.

**Conclusions:**

To improve antibiotic use in children under 5 years of age in Northern Tanzania, the precarious situation that women often find themselves in as they access treatment for their sick children needs to be taken into consideration. It is necessary to improve structures, including the healthcare system, socioeconomic inequalities and promoting gender equality both in the household and in the public arena to reduce misuse of antibiotics. Meanwhile, equipping community health workers to support Tanzanian women in appropriate healthcare seeking for their children, may be a feasible target for intervention.

**Supplementary Information:**

The online version contains supplementary material available at 10.1186/s13756-022-01169-w.

## Background

Antibiotics are vital in the treatment of bacterial infections in children. Meanwhile, all antibiotic use in humans, animals or agriculture is promoting the development of antimicrobial resistance (AMR) [1]. This represents a serious threat to the global achievements in child health thus far [2]. Low- and middle-income countries (LMICs) are particularly vulnerable to AMR development due to weak pharmaceutical governance, lack of sanitation and limited access to safe water [3]. In recent years, antibiotic use in LMICs has increased rapidly [4] and studies from Moshi in Northern Tanzania have revealed high use of antibiotics in children even in cases of minor symptoms indicating a common cold [5, 6]. Although the primary care-givers in LMICs play a key role in accessing and administering antibiotics for their sick children, their perspective is largely unknown. In this qualitative study we have sought to describe mothers’ experiences of antibiotic use in under-five children, thus contributing to the growing body of work seeking to understand AMR as a biosocial issue [7–9].

## Methods

### Study design and approach

This qualitative study was performed with a phenomenographic approach. Phenomenography was first developed by Marton through studies of learning in higher education [10, 11] and has since been applied to describe experiences and conceptions of both healthcare workers and recipients [12–14]. This approach is particularly suitable when aiming to describe the qualitatively different ways in which a group of people experience and comprehend different phenomena in the world around them. Interviews, individually or in groups, are common as a means of data collection in phenomenography [11]. In this study, focus group discussions (FGDs) were used, this being a form of structured discussions performed in a non-threatening environment which encourages in-depth discussions through group interaction [15].

### Study setting

The study was part of a larger study including both prescribing primary healthcare workers (HCWs) and parents or guardians of children under 5 years in the community of Moshi Municipal (urban) and Moshi District (rural) Councils, in the Kilimanjaro region of Northern Tanzania. Results from in-depth interviews with prescribing primary healthcare workers (HCWs) have been described previously, together with the the local healthcare system and the general demographics of the region [16]. The main income for inhabitants in urban Moshi is from farming, trading in primary produce, small retail outlets and tourism. In rural Moshi the key economic activities are farming and trading in primary produce. Poverty incidence in Kilimanjaro region was among the lowest in the country in 2018. However, approximately 8–21% of the population was still estimated to live below the national poverty line (TZS 49,320 per adult per month) [17]. Almost all families in Tanzania attend routine healthcare for their children which includes immunization, growth monitoring and sometimes health education [18]. The conducting of FGDs with parents or guardians at the premises of a primary healthcare facility was therefore considered to be viable. The facilities are presented in Table 1.

**Table 1:** Characteristics of the focus groups

	Participating mothers					Health facility			
Focus group	No. of participants	Age, years	Mean no. of children	Education^a^	Marital status^b^	Level	Owner	Area	District^c^
1	7	20–33	1.7	P (1) S (5) U (1)	M (6) S (1)	Dispensary	Public	Urban	MU
2	7	19–45	2.3	P (5) S (2)	M (4) S (3)	Health Centre	Public	Urban	MU
3	7	22–35	2.1	P (3) S (3) U (1)	M (6) S (1)	Health Centre	Private	Urban	MU
4	7	19–40	1.9	P (5) S (2)	M (6) S (1)	Health Centre	Public	Urban	MU
5	7	20–37	2.3	N (1) P (4) S (2)	M (7)	Dispensary	Public	Urban	MU
6	6	19–33	2.3	P (2) S (3) U (1)	M (6)	Dispensary	Public	Semi-urban	MU
7	6	19–39	1.8	P (5) S (1)	M (6)	Health Centre	Public	Rural	MR
8	7	21–39	1.9	P (3) S (2) U (2)	M (3) S (4)	Dispensary	Catholic organisation	Rural	MR

^a^No education (N), Primary school (P), Secondary school (S) or University degree (U)

^b^Married (M); Single (S)

^c^Moshi urban (MU) or Moshi rural (MR)

### Data collection

Data collection was conducted during September–November 2019. The research team (CM, EM, GN, ME and RM) was appointed and trained as previously described [16]. An interview guide (in English) was developed by ME, ML and RM and translated into Kiswahili by CM, EM, GN and RM. In Kiswahili, the English term, ‘antibiotics’ (i.e., anti-bacterial substances) has been adopted into the language; therefore the term, ‘antibiotics’ was also used in the translated interview guide (Additional file 1). A suitable day to conduct the FGD was agreed upon between the heads of the healthcare facilities [16]. Participants were selected by convenience sampling; parents or guardians attending with their children were approached face-to-face and were invited to participate by a local nurse who had been briefed about the purpose of the study. Although male care-givers were accepted, no men were present to be included. The participants were given further oral and written information (in Kiswahili) concerning the study by the team and were asked to give their written consent to partake (Additional file 2). Each participant was asked questions concerning age, marital status, number of children and education. In total, 54 mothers were included in eight different focus groups (Table 1). Two mothers included in focus group 2 and one in focus group 6 left during the discussion due to their child being upset. The FGDs were held in a private room and the participants were all sitting in a circle together with the moderator and light refreshments were offered. The initials of each participant were placed in front of them and the moderator referred only to these to ensure confidentiality. During the FGD only the participants with children and the researchers were present. The FGDs were moderated by CM, EM and RM with assistance from 2 to 3 team members (including GN and ME). Before describing their experiences of antibiotic use in under-five children, the participants were asked to identify and give examples of antibiotics. After the first FGD the team discussed and updated the interview guide. All FGDs were digitally audio recorded (Olympus Dictaphone) and one team member was taking notes as a complement. Each FGD lasted between 30 min and 1 h.

### Data management and analysis

All FGDs were transcribed verbatim by a team member (CM, EM, GN) who had been present at the FGDs. Translations were undertaken by clinicians at Kilimanjaro Christian Medical Centre, Moshi, Tanzania who were fluent in both Kiswahili and English. Part of the translated material was back-translated by FM and RM to ensure quality and conformity of the translations. NVIVO 12 software (QRS international) was used for data management. Data analysis was performed according to the phenomenographic approach and followed the steps as described by Sjöström and Dahlgren [19]. ME was mainly responsible for coding and presentation of data to the co-authors (FM, ML, RA, RM and SS) and the results were discussed and adapted at regular seminars. A neutral co-examiner tested the results to ensure correct data analysis through assigning uncoded quotations to these categories. Agreement was almost unanimous between the authors and the co-examiner.

## Findings

**Fig. 1:**
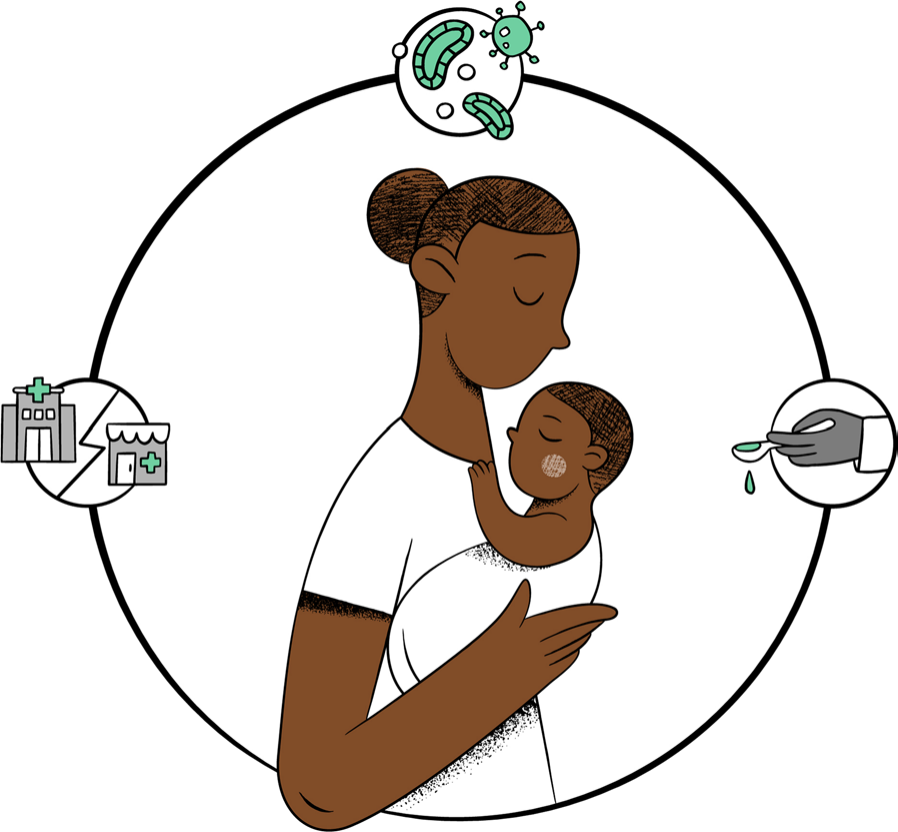
The perspective of the caring mother: themes that emerged in the analysis. 1. Conceptions of disease and antibiotics (top); 2. Conceptions of accessing treatment (left); 3. Conceptions of administering medication (right)

During analysis of the FGDs, three conceptual themes emerged (Fig. 1), with associated categories (Table 2).

**Table 2:** Themes and categories that emerged in the analysis

*1. Theme*	*Conceptions of disease and antibiotics*
1.1 Category:	Causes of common childhood diseases
1.2 Category:	Antibiotics are a remedy for common symptoms
1.3 Category:	Antibiotics are used to treat specific diseases
1.4 Category:	Antibiotics are a universal treatment
*2. Theme*	*Conceptions of accessing treatment*
2.1 Category:	Home treatment is used before seeking healthcare
2.2 Category:	Healthcare facilities are trustworthy
2.3 Category:	Healthcare facilities may not be accessible or satisfactory
2.4 Category:	Pharmacies are cheap and convenient
*3. Theme*	*Conceptions of administering medication*
3.1 Category:	Understanding and receiving instructions
3.2 Category:	Storing and re-using the medication
3.3 Category:	Adherence to prescription

## Theme: conceptions of disease and antibiotics

The first theme relates to conceptions of the causes of common childhood diseases and why antibiotics were used. In describing disease, the mothers used the term ‘magonjwa’, the Kiswahili word which refers to any disease, and is not specific for infectious disease. The mothers frequently referred to ‘dawa’ which is a term for any biomedical drug.

### Category: causes of common childhood diseases

This category comprises a broad variety of statements with explanations for common childhood diseases. Origins for symptoms or infections were often found in the natural environment of the child.

*“Mafua (flu) is caused by hali ya hewa (weather) and wind (…) [it] can also be caused by dust or harufu kali (strong smell) used by the mothers (…) Or when the child eats something contaminated.”* (FGD 7).

Cough was a common symptom, either described with the term, ‘kifua’ (literal translation ‘chest’, also used for any chest infection) or ‘kikohozi’, perceived to have different causes such as teething, dust, cold or swallowing infected amniotic fluid at birth. The mothers also described behaviour that could lead to disease, such as letting the child play in dirty water or inadequate hygiene. At times, the mothers described disease-causing microbes using the general terms, ‘mdudu’ (bug) or ‘vijidudu’ (germ). However, in no statement was virus mentioned as a cause of disease. Upon probing, a mother described bacteria as follows: *“To my understanding these [bacteria] are wadudu (bugs) who live in water or foods that are not covered well.” *(FGD 3).

### Category: antibiotics are a remedy for common symptoms

Statements in this category reveal that the mothers perceived antibiotics as a remedy for common symptoms including fever, cough and diarrhoea. Antibiotics were, in these cases perceived to be similar to paracetamol or cough syrup. Cough was commonly treated with antibiotics, as shown in the following statement:

*“There are many types of kikohozi (coughs), the one for kifua kikuu (pulmonary tuberculosis) and the kikohozi cha kawaida (normal cough). The kawaida (normal) is the one we use antibiotics.”* (FGD 7).

Some mothers described previous use of non-antibiotic remedies for common symptoms such as infant colic, who were now using antibiotics instead:

*“(…) this matumbo (intestinal pain) is common [in infants] (…) as for my children I gave them amoxicillin. Previously we gave them Gripe water [containing sodium bicarbonate/herbs] but this has been banned and [now] I usually buy these dawa (medicines) from the pharmacy.”* (FGD 7).

### Category: antibiotics are used to treat specific diseases

This category illustrates conceptions of antibiotics as a treatment for some specific diseases. Antibiotics were, in such cases perceived as being different to symptomatic treatment although statements show that they might not be perceived as specific for bacterial infections. *“Antibiotics are dawa (medication) prescribed to you if, for example, you have tested positive for malaria or amoebic diseases and they are different from paracetamol.” *(FGD 3).

However, in some statements, antibiotics were described as being different to treatments for fungii or parasites.

*“I think malaria [is not treated with antibiotics] because mostly when one has malaria, mseto (a mixed anti-malarial drug) or Fansidar (Sulfadoxine–Pyrimethamine) is prescribed and the person is cured. I have never seen a person with malaria who had antibiotics prescribed for them.”* (FGD 1).

### Category: antibiotics are a universal treatment

The fourth category illustrates the conception of antibiotics as a universal treatment, used to treat or prevent most symptoms or diseases in children.

*“It [antibiotics] is a dawa (medicine) that treats many diseases, even if you have malaria, because it contains so many ingredients. If the child has cough and flu the antibiotics treats them all.” *(FGD 7).

According to the statements, antibiotics were also perceived as protecting the body or strengthening the children’s immune system. *“Because most times it [antibiotics] is dawa yenye kinga (protective medication) in the body and they are not so strong for children under the age of 5 years.”* (FGD 2).

Few statements show little or no conception of antibiotics but did, in contrast describe antibiotics as ‘strong’. Nevertheless, antibiotics were often perceived as a comprehensive treatment with little or no harmful side-effects.

## Theme: conceptions of accessing treatment

The second theme involves conceptions of how the mothers access treatment for their sick child, including possible barriers and preferences. The mothers often used ‘hospitali’ (hospital) to describe any primary healthcare facility or district hospital; ‘daktari’ (doctor) was used to refer to HCWs regardless of education level and ‘duka la dawa’—or sometimes ‘pharmacy’—was used to refer to any level of community pharmacy.

### Category: home treatment is used before seeking healthcare

The statements in this category revealed that, before seeking care for their sick child, some mothers were accustomed to treating their child at home, primarily to reduce fever or pain. Some mothers administered paracetamol at home, whilst the use of traditional or herbal medicine was not mentioned. The mothers agreed that, if there was no improvement the child was brought in to be seen by an HCW.

*“If we give a child dawa (medicine) without consulting a daktari (doctor), if it takes two days without recovery, we go and tell the daktari that we have given the child this dawa (…) so as the daktari can know which test to take and what the real problem is.”* (FGD 2).

Several statements showed that the mothers have sought medical advice from a nurse or experienced mother in the neighbourhood regarding the treatment of their sick child.

*“I am lucky, I live very close to a nesi (nurse) so when the child gets sick, I go to her even at night. She gives me dawa (medicine) and advises me that if the child does not get better, to take him to the hospitali (hospital) the next day.”* (FGD 7).

### Category: healthcare facilities are trustworthy

This category comprises conceptions of healthcare facilities as trusted institutions and HCWs as the most qualified to assess the sick child. According to the statements, the main reasons why the mothers preferred to seek care at a healthcare facility were as follows: the child would undergo a physical examination and treatment and/or advice would be provided. When asked if it would be a problem if the HCW did not prescribe medicine, a participant explained:

*“I will not agree if [the child] has not been checked for temperature; to me it will be a problem. (…) I will agree with him as long as investigations have been taken and knowing the status [of the child] is progressing well.”* (FGD 2).

Thus, provided that the HCW performed a thorough assessment of the child which revealed no need for prescribed treatment, the mothers often felt reassured. Some also shared experiences of adverse events when a child had not been seen by an HCW. Such experiences enforced the perception of the healthcare facilities as being the safest option:

*“There is also our neighbour whose child died [because] the child was sick and the parents did not take her to the hospitali (hospital), instead they bought dawa (medicine) from the shop. So, when my child is sick even at night, I go to the hospitali (…) I never buy dawa from the shops.”* (FGD 5).

### Category: healthcare facilities may not be accessible or satisfactory

The mothers faced several constraints in accessing treatment at the healthcare facilities and, on occasions the visits did not meet with their expectations. Although public healthcare facilities were supposed to provide free treatment for children under 5 years, there could be hidden costs such as transportation, registration and paying for medicines that may be out of stock. However, such costs were often not apparent to their husbands who provided the money.

*“(…) they say that treatment for children less than 5 years is free (…) it is true that the consultation is free, but then the daktari (doctor) tells you we do not have this dawa (medicine), go and buy in the duka la dawa (pharmacy) (…) you have to go and ask for money from the man and he tells you ‘I don’t have money, take this bus fare, go and get the child treated’. You tell [him] but he insists that treatment is free for children.”* (FGD 3).

Furthermore, some statements described situations in which the mothers were not satisfied with the care provided at the healthcare facility. These included inadequate soothing of a child’s cough which may then have led them to consult the pharmacy staff. Others had experienced long waits at the healthcare facility, without receiving care for their sick child or had encountered disrespectful treatment from the HCWs.

*“Sometimes there are few staff at the facility; you get there early in the morning and wait till evening without service! For example, yesterday we waited for a long time (…) a child fainted due to seizures because the fever was severe.”* (FGD 2).

### Category: pharmacies are cheap and convenient

This category describes reasons for approaching pharmacies in the first instance to obtain treatment for the sick child. Firstly, accessing treatment at the pharmacies was perceived as being cheap. Furthermore, the mothers found a justification for not attending the healthcare facility when buying medicines which had been used or prescribed previously. Whilst some mothers asked for a specific antibiotic, others consulted the pharmacy staff.

*“Sometimes due to distance [to the healthcare facility] (…) I take the child to the pharmacy and explain how the child is coughing. Because the pharmacist has studied, he gives dawa (medication).”* (FGD 1).

Thus, pharmacy staff were trustworthy. In addition, the pharmacies were also perceived as being more accessible as they were situated closer to the families’ homes and had longer opening hours. However, some pharmacy staff did not provide medicine unless the child had been seen by an HCW.

*“There are times when we go to the duka la dawa (pharmacy) and if we tell them we haven’t attended the hospitali (hospital) we are denied to get the dawa (medicine). They often advise young children to start at the hospitali.”* (FGD 4).

## Theme: conceptions of administering medication

The third theme relates to conceptions concerning how medicines were, or should be handled and administered to children.

### Category: understanding and receiving instructions

The first category involves perceptions of how and by whom instructions were given to the mothers when prescribing a medicine for her child. The mothers largely agreed with the following statement: *“The daktari (doctor) just writes the prescription form and you get all other information at the dispensing window.”* (FGD 2).

In general, the mothers claimed that the instructions provided were easy to understand and some stressed the importance of understanding the instructions to avoid adverse events. However, others were hesitant in asking the HCW to clarify instructions if these were difficult to understand and/or contradictory.

*“To be honest I [personally] do not ask questions. The daktari (doctor) writes the prescription, [but] I don’t know how to read so I don’t know anything, even English I don’t know… Someone else when you ask him [the doctor] gets angry. Mostly amoxicillin is the one I can read [identify], even the day my child was given 25 injections I did not ask.”* (FGD 8).

According to the statements, fear of being rebuked by the HCW if raising questions or concerns has led to a lack of understanding about the prescribed treatment.

### Category: storing and re-using the medication

This category comprises conceptions on how medicine for children should be stored and possible re-usage in subsequent disease episodes. The mothers were often advised by the HCWs on these issues.

*“For example, you buy dawa (medicine) after being investigated and found with a problem, the nurse tells me to use the dawa, and to keep the remaining one safe and it can be used again when the symptoms recur.”* (FGD 3).

Although the mothers knew less about differences in mode of action between the different medicines, they generally perceived medicines which were ready to use (mainly paracetamol) as being eligible for re-usage whilst those diluted with water (mainly antibiotics) should be discarded. However, some mothers did not re-use medicines to avoid the risk of using expired medicines or the risk of re-using antibiotics inappropriately for a different disease.

*“It depends what kind of dawa (medication), if it is liquid, it means after 7 days it will expire (…). But be careful, because the baby may have other symptoms which are not like the ones before, so it will be better to go to the hospitali (hospital).”* (FGD 8).

### Category: adherence to prescription

The last category includes statements describing experiences of adhering to the prescribed treatment course and any perceived motives or barriers to doing so. Whilst most mothers stated they would finish the prescribed course, some did not as their children often became well before the end of the course. When explaining why it is important to adhere to the prescribed interval, a participant stated: *“If you don’t give dawa (medication) on a prescribed time it will make the mdudu (bug) acting like kazimia (fainting). When he wakes up the problem starts again.”* (FGD 1).

Thus, according to the statement, not adhering to prescription may lead to failure to eradicate the pathogen and consequently disease recurrence. However, the mothers experienced medicine administration at the prescribed hours as a challenge, due to their daily chores.

*“For example, you are required to give dawa (medication) at 6:00am. Instead, I struggle to wash my dishes and finish my work so I can give the dawa to my child. [But] the time I have been prescribed by the daktari (doctor) to give the child dawa has already passed!”* (FGD 3).

## Discussion

The detrimental effects of increasing AMR which disproportionally affect LMICs require an understanding of what drives inappropriate antibiotic use in the local context [20]. Preschool children undergo a large number of infections yearly and studies from low malaria endemic areas in Tanzania suggest most acute febrile illnesses in children are, in fact viral [21, 22]. However, determining when a child needs professional healthcare or treatment and finding ways to seek help still pose a considerable challenge for primary care-givers in LMICs where child mortality remains high. We performed FGDs with mothers in urban and rural Moshi, Tanzania on their experiences of antibiotic use in children. Below we discuss our findings in the context of previous studies addressing antibiotic use from a social science perspective.

The ambition of the Tanzanian government is to provide free healthcare for children under 5 years. However, women in our study disclosed hidden costs that posed barriers to healthcare seeking. These includes travel, long waits at the healthcare facility (leading to loss of possible daily income), dubious costs for registration and purchase of prescribed medicines from pharmacies. These barriers were similar to those found in a qualitative study on mothers’ healthcare seeking behaviour in relation to childhood pneumonia symptoms, carried out in urban Moshi [23] and a qualitative study on self-medication of antibiotics among pharmacy clients in Maputo city, Mozambique [24]. In the face of severe resource constraints, reducing some of the described costs by, for example purchasing antibiotics from a nearby pharmacy may indeed be viewed as a ‘rational’ behaviour. Further, qualitative studies of community use of antibiotics in urban slum areas of Kinshasa, Democratic Republic of Congo [25] and rural central Ghana [26] also revealed travel and out-of-pocket payments at healthcare facilities as major barriers for healthcare seeking, leading to widespread self-medication of antibiotics. In contrast, in rural South Africa where primary healthcare provided by the government is free and prescription-only antibiotic sales enforced, self-medication with antibiotics has been low [20, 27]. Poor supply chains are a common challenge to the healthcare system in Tanzania causing a shortage of medicines including antibiotics. This may have affected prescribing practices at public facilities as shown by our previous study [16] where poor families were more likely to be prescribed antibiotics that were available at the facility free of charge rather than those most suitable for the infection. Low socioeconomic status may therefore be a determinant for inappropriate use of antibiotics in Tanzanian children due to both self-medication and inadequate access to antibiotics at public healthcare facilities. However, for some mothers it was not an absolute deficit of money but rather restricted access to household money and lack of support from the husband that led them to primarily access treatment at the pharmacy. The way in which gender structures affect AMR is currently an understudied area [28]. Nevertheless, there is evidence in the literature that points to women’s and girls’ empowerment leading to better health and development outcomes [29]. Our results suggest that promoting gender equality and female empowerment will contribute to more appropriate healthcare seeking for children. This may thus lead to less irrational use of antibiotics.

In the FGDs, some women stated that they would be disappointed if not prescribed an antibiotic by the HCW when seeking healthcare for their sick child. This expectation may be in line with what Denyer-Willis and Chandler [7] describe as the ‘pharmaceuticalisation’ of healthcare. In resource limited settings, this is reflected in the use of algorithms resulting in the provision, or not of specific medicines rather than a clinical assessment. However, as the discussion developed, the women explained that what they were actually seeking was a proper assessment of the sick child. Thus, the expectation of antibiotics may be seen as a compensation for lack of care, whilst the mother’s greater wish was for a clinical assessment and reassurance [30]. Likewise, parents in high-income countries are mainly concerned with receiving a diagnosis and being reassured that the child is not severely ill, although this may be misinterpreted by clinicians as expectations of antibiotics [31, 32]. In our previous interviews, HCWs indeed expressed the perception that mothers wanted antibiotics prescribed for their children [16]. This illustrates similar ‘dissonant views’ between parents and HCWs in Northern Tanzania [32]. The need for addressing the unequal power relationships between mothers and HCWs was shown in some citations where the women expressed a fear of being rebuked by the HCW if raising questions or concerns. There is a need for targeted communication training for HCWs on the importance of sensitively exploring the real concerns and/or expectations of parents and providing appropriate information about treatment. However, limited time for each consultation due to high demand may cripple such interventions. This highlights that continued strengthening of the healthcare systems in LMICs including support for HCW in providing respectful healthcare for mothers and children [33], are crucial for improving antibiotic stewardship [8].

Our study confirms that community pharmacies are perceived as forming an integral part of the healthcare system in Tanzania [34]. Although mothers preferred to seek care at healthcare facilities for their children, pharmacy staff were also trusted and were perceived as being ‘educated’. Availability of antibiotics from Accredited Drug Dispensing Outlets (ADDOs, also referred to as type II pharmacies) has been a strategy to limit usage of local retail drug shops and improve access to effective medicines in rural and peri-urban areas of Tanzania [35]. Whilst type I pharmacies are supposed to be run by a registered pharmacist, type II pharmacies can be run by anyone who has completed a 5 weeks’ training course. However, while antibiotics are classified as prescription-only drugs in Tanzania, the majority of community pharmacies do not adhere to this upon customer request [35–37], possibly due to the impact of market forces [35]. In this study, women primarily attending a pharmacy described either having requested a specific antibiotic they had previously used or having consulted the staff. In line with previous studies [35–37], only a few statements described pharmacy staff refusing to sell antibiotics without a prescription.

Throughout the FGDs, antibiotics were frequently combined inappropriately with other drugs such as paracetamol. The terms for antibiotics, bacteria and viruses have been adapted from English and brought into the Kiswahili language but were rarely used by women in our study. Instead, the Kiswahili terms for medicine (dawa), bug (mdudu) or disease (magonjwa) rather than infection, were used although these terms have a more generalised meaning. The conception of antibiotics as a universal treatment for sick children was apparent and may be influenced by this generalized language. The perception of antibiotics as capable of curing almost any disease has been previously described in LMICs [20, 38]. However, the conception of antibiotics as ‘strong’ was not prevailing in our data, rather the emphasis was on antibiotics as a comprehensive treatment with few or no harmful side-effects. As antibiotics have become part of the infrastructure in LMICs [39], their familiarity may also have reduced their perceived ‘strength’. Whilst a deeper knowledge of the effect of antibiotics was lacking among the mothers, the practical knowledge on administering and storing antibiotics was seemingly better. In cohesion, a recent study from South Africa concluded from observations that most advice given by HCWs was pragmatic, whilst not including information on self-care or antibiotic resistance due to the limited time spent with each patient [40]. The few women in our study who had heard of antibiotic resistance perceived that development of resistance was associated with not completing the prescribed course or frequent use of antibiotics. These were also the two most common beliefs among the public as was found in a systematic review of studies mainly performed in high-income countries [41]. Antibiotic resistance was described by the mothers as a concern for, or within the individual rather than a public health problem, similarly as expressed by the HCWs in our previous study[16], by patients and prescribers in South Africa [40] and adult community members in rural, Southern Mozambique [42].

Appropriate healthcare seeking is important for the safety of patients and the efficacy of healthcare providers. In children, safe home practices with non-antibiotic remedies for minor illnesses should be promoted together with how to recognize danger signs to not delay healthcare seeking in serious situations. In line with previous findings [23], this study shows that mothers who felt unsure have been seeking out an experienced nurse or mother in the neighbourhood for advice as to whether the child needed professional care. This resembles the role of the community health workers (CHWs) in Tanzania, trained volunteers who work at the frontline of communities [43]. In recent years, CHWs are being integrated into the healthcare system to compensate for the country’s critical shortage of educated HCWs [43, 44]. A recent report on AMR and social science identifies three areas to address antibiotic use, namely practice, structures and networks [8, 45]. Whilst practice has generally been assumed to be improved by increased public awareness of AMR, social scientists have suggested that in LMICs precarity (lack of predictability or security) and weak social support are more significant determinants of antibiotic use [46–48]. Although women in this study had very limited knowledge of AMR, these recent findings suggest educational activities will not be sufficient to improve their use of antibiotics. Furthermore, structures such as healthcare systems, market driven antibiotic sales, income and gender inequalities have clearly been affecting antibiotic use in children in Northern Tanzania. However, the increase in AMR requires action that cannot wait until these issues are fully resolved. Thus, a feasible way forward could be equipping networks, involving an increased presence of CHWs to support women in appropriate healthcare seeking for their children and in consequence, advocating for the prudent use of antibiotics. A promising area of intervention is providing CHWs with mobile health solutions (mHealth) to improve their performance and efficacy [49].

## Strengths and limitations

Although all women in the FGDs were encouraged to share their unique experiences, older women with several children were, in general more confident in speaking compared to the younger women. This may be a reflection of added experience on the topic, but also a consequence of sociocultural norms [15]. However, if participants had been divided into different FGDs according to age, fewer sites could have been included resulting in a loss of variability. Nonetheless, the fact that some FGDs were being observed by a western, female researcher did not seem to affect the openness of the discussions and this may be attributable to the local researchers taking the leading role in interacting with the participants and moderating the FGDs in the participants’ native language. The majority of focus groups (6/8) were carried out in an urban or semi-urban area. The voices of women in this study were thus mainly from urban areas or rural areas with reasonable access to healthcare. Future studies should be undertaken in areas with more restricted access to healthcare exploring how this affects healthcare seeking for children in relation to antibiotics. It should be noted that the FGDs were carried out before the Covid-19 pandemic, if this have had an effect on the conceptions of disease and antibiotics need to be addressed by future studies.

## Conclusions

This study has portrayed the experiences of mothers in Northern Tanzania on antibiotic use in their under-five children, revealing the precarious situation that largely affects the women as they access treatment and administer medication to their sick children. Therefore, we suggest improving antibiotic use in children under 5 years of age in this area requires more than raising public awareness of AMR. Rather, there needs to be a focus on improving structures, including the healthcare system, socioeconomic inequalities and promoting gender equality both in the household and in the public arena to reduce misuse of antibiotics. Meanwhile, equipping networks involving an increased presence of CHWs to support Tanzanian women in appropriate healthcare seeking for their children may be a feasible target for intervention.

## Supplementary Information

**Additional file 1**. Focus group guide for parents or guardians (English/Kiswahili)

**Additional file 2**. Participant Information Sheet and Consent forms (English/Kiswahili)

## Data Availability

Anonymised datasets analysed and generated during this study are available from the corresponding author upon reasonable request.

## Abbreviations

CHW: Community health worker
FGD: Focus group discussion
HCW: Healthcare worker
LMIC: Low- and middle-income country
