# Supplementary material for: Antibiotic use in children under 5 years of age in Northern Tanzania: a qualitative study exploring the experiences of the caring mothers
Source: Antimicrob Resist Infect Control. 2022 Nov 3;11:130. doi: 10.1186/s13756-022-01169-w (PMC9630810; doi:10.1186/s13756-022-01169-w)
Supplement: Supplementary file 1 — Additional file 1. Focus group guide for parents or guardians (English/Kiswahili) [file 13756_2022_1169_MOESM1_ESM.pdf]

Focus group guide for parents or guardians

1. **Screening question**

Do you know what an antibiotic is? Can you mention any common antibiotic?

2. **Opening question**

Can you please describe your experiences of antibiotic use in children under 5 years of age?

3. **Follow up questions**

3.1 Do you know what sort of illnesses antibiotics treat in children?

3.1.1 Do you know what sort of illnesses antibiotics **do not** treat in children?

3.1.2 What is **the cause** of those illnesses?

3.2 Where do you go if your child is sick or you think he/she needs an antibiotic? **Why** do you go to the clinic/pharmacy?

3.2.1 Is there any type of illness when you can administer antibiotics to your child without a prescription from a clinician?

3.2.2 Have you experienced going to see a clinician with your child and **not** receiving an antibiotic when you felt you should have? Can you please give examples? What did you do?

3.2.3 Do you keep antibiotics at home in case you need them for your child?

3.2.4 What would encourage you to seek the help of a clinician instead of the pharmacist in case your child is unwell?

3.3 When dispensed an antibiotic what kind of instructions do you receive?

3.3.1 Do you find them difficult/easy to understand? Can you please give examples?

3.3.2 When do you stop giving an antibiotic to your child? When the child is well or when the course is finished?

3.4 Have you ever experienced giving an antibiotic to your child, but the child did not improve? Can you please give examples? What did you do next?

3.5 Have you ever heard of bacteria becoming resistant to antibiotics?

Focus group guide for parents or guardians

1. **Maswali ya awali**

   Unajua antibiotiki ni aina gani ya dawa? Unaweza kutaja baadhi ya dawa za antibotiki?

2. **Utangulizi**

   Unaweza kunieleza uzoefu wako dhidi ya dawa za antibiotiki kwa Watoto chini ya miaka mitano?

3. **Maswali ya kufuatilizia**

   3.1. Je unajuwa magonjwa ambayo yanatibiwa kwa kutumia antibiotiki?

   3.1.1. Je unajuwa magojwa ambayo dawa za antibiotiki haziwezi kutibu kwa watoto?

   3.2. Je unaenda wapi kama mtoto anaumwa na unahisi anahitaji kutibiwa na antibiotiki?

   3.2.1. Je kuna ugojwa wowote ambao unaweza kumpa mtoto antibiotiki bila kuandikiwa na daktari?

   3.2.2. Je ushawai kwenda kumuona daktari na mtoto wako lakini hukupata dawa ya antibiotiki kama wewe ulivyokuwa unategemea? Una mfano wowote? Je ulifanyaje?

   3.3. Je unapotata antibiotiki unapewa maelezo gani?

   3.3.1. Je yale maelezo ni magumu/rahisi kufwatilia? Unaweza kunipatia mfano?

   3.3.2. Je ni lini unamwachisha kumpa mtoto antibiotiki? Je ni wakati amepona au ni wakati dozi imeisha?

   3.4. Je umeshawahi kumpatia mtoto dozi ya antibiotiki lakini hakupata nafuu? Unaweza kutoa mfano? Je ulichukuwa hatua gani?

   3.5. Je umeshawahi kusikia kuhusu bakteria kuwa sugu kwenye dawa ya antibiotiki
