# Supplementary material for: Antibiotic use in children under 5 years of age in Northern Tanzania: a qualitative study exploring the experiences of the caring mothers
Source: Antimicrob Resist Infect Control. 2022 Nov 3;11:130. doi: 10.1186/s13756-022-01169-w (PMC9630810; doi:10.1186/s13756-022-01169-w)
Supplement: Supplementary file 2 — Additional file 2. Participant Information Sheet and Consent forms (English/Kiswahili) [file 13756_2022_1169_MOESM2_ESM.pdf]

UNIVERSITY OF
GOTHENBURG

Participant Information Sheet

This informed consent form is for parents or guardians with children under 5 years that we are inviting to participate in research on experiences of antibiotic use in children under 5 years of age.

I am a researcher from Kilimanjaro Christian Medical University College (KCMUCo) in Moshi, supervised by Prof. Sia Msuya and Lecturer Rose Mwangi representing the department of Community Health. The studies are made in collaboration with the University of Gothenburg, Sweden.

Infections are common in children under 5 years of age. This proposes challenges for all parents of guardians when faced with the question of how serious the present symptoms are and when it is appropriate to seek care and give a specific treatment.

You as a parent/guardian are invited to participate in the study through taking part in a group discussion held at the health clinic you are currently attending since you are the sole taker of the child, your experience will inform our findings.

You may not receive any payment from participating and there may not be a direct benefit for you. However, the results from this study will benefit the society as a whole in the future. You and your child's participation will be kept confidential. Neither your or your child's name will be recorded. Instead, a unique identification number will be used. We appreciate if you answer all the questions honestly and to the best of your knowledge. If there is any question you do not want to answer you are free to refuse. You are also free to ask any questions.

Participating in this study may take some of your time (less than an hour), however, you are free to withdraw from this study at any time, and you do not need to give a reason. If you

decide to withdraw, you can inform the interviewer/study coordinator and no new information will be collected about you or your child, other than that needed to keep track of your withdrawal.

Your participation in this research is entirely voluntary. You are free to participate or not to participate in this study. You or your child's right to receive care in this facility will not be affected if you choose not to participate.

Contact details

Local Principal investigator:
Prof. Sia E. Msuya, MD, PhD
Director Institute of Public Health
Kilimanjaro Christian Medical University College
Box 2240 Moshi Kilimanjaro Tanzania
Phone: + 255 784 405619
Email: siamsuya@hotmail.com

Principal investigator:
Matilda Emgård, MD, PhD-student
Department of Infectious Diseases
Institute of Biomedicine
University of Gothenburg
Gothenburg Sweden
Email: matilda.emgard@gu.se

National Health Research Ethics Sub-Committee (NatHREC):
National Institute for Medical Research
P.O. Box 9653, Dar es Salaam, Tanzania
Tel.: +255 22 2121400
Mobile: +255 758 587885
Hotline: +255 22 2130770
Email: ethics@nimr.or.tz / nimrethics@gmail.com

UNIVERSITY OF GOTHENBURG

Consent Form

The purposes of this study and the study procedures, risks and benefits have been explained to me. I have been allowed to ask questions and my questions have been answered to my satisfaction. I have been told that I may contact the KCMC Ethics committee if I have questions about my rights as a research subject.

I confirm that I have read the participant information sheet or that it has been read to me. I understand that my participation is voluntarily, and that I am free to withdraw at any time, without my legal rights being affected. I have been informed that I will be given a copy of this consent form for my record. *I agree to take part in this study.*

Name of participant…………………………………… Signature ……………………..

Date…………………………..

Name of researcher taking consent…….................... Signature …………….….......

Date…………………………..

Witness (if applicable)…………………………….…..Signature……………………...

Date……………………………

Fomu ya Mshiriki

Fomu hii ni ya kuomba ridhaa kwa wazazi au walezi wenye watoto chini ya umri wa miaka 5 watakaoshiriki katika utafiti wa kuangalia uzoefu wao kuhusu matumizi ya antibotiki kwa watoto walio na chini ya miaka mitano

Salaam Mimi ni mtafiti kutoka Chuo Kikuu cha KCMC huko Moshi, kinachosimamiwa na Dk. Sia Msuya na Mwalimu Rose Mwangi anayewakilisha Idara ya Afya ya Jamii. Huu utafiti umefanyika kwa kushirikiana na Chuo Kikuu cha Gothenburg, Sweden.

Homa na dalili za magonjwa zinatokea mara kwa mara kwa watoto chini ya umri wa miaka 5. Hivyo imeleta changamoto kwa wazazi au walezi wakati wanakabiliwa na maswali kwamba ni kwa kiwango gani cha homa ambacho mzazi/mlezi atatakiwa kumwona daktari kwa matibabu

Wewe kama mzazi / mlezi unaalikwa kushiriki katika utafiti huu kwa kushiriki katika mjadala wa kikundi ambao utafanyika katika kituo cha afya unachohudhuria sasa kwa kuwe wewe ni mlezi/mwangalizi mkuu wa mtoto na uzoefu wako utatusaidia.

Hutapewa malipo yoyote kwa kushiriki kwako na inawezekana usipate faida ya moja kwa moja kwako. Hatahivyo, matokeo ya utafiti huu utafaidisha jamii kwa ujumla hapo baadae. UShiriki wakop utakuwa wa usiri na jina lako halitarekodiwa ila itatumika namba ya siri kama utambulisho. Tutashukuru kama utajibu maswali yote kwa uaminifu na kwa jinsi unavyoelewa. Kama kuna swali hutaki kujibu uwe huru kutojibu au kukataa kujibu. Pia uko huru kuuliza maswali.

Ushikiri wako katika utafiti huu ni wa hiari kabisa. Uko huru kushiriki ama kutokushiriki katika utafiti huu. Uko huru kujitoa katika utafiti huu muda wowote ule na hauiotaji kutoa sababu.

Kama utaamua kujitoa, unaweza kumfahamisha mdodosaji au msimamizi wa utafiti na hamna habari mpya zozote zitachukuliwa zaidi ya zile zinazoitajika kuweka rekodi ya kujitoa kwako.

Contact details

Local Principal investigator:

Prof. Sia E. Msuya, MD, PhD

Director Institute of Public Health

Kilimanjaro Christian Medical University College

Box 2240 Moshi Kilimanjaro Tanzania

Phone: + 255 784 405619

Email: siamsuya@hotmail.com

Principal investigator:

Matilda Emgård, MD, PhD-student

Department of Infectious Diseases

Institute of Biomedicine

University of Gothenburg

Gothenburg Sweden

Email: matilda.emgard@gu.se

National Health Research Ethics Sub-Committee (NatHREC):

National Institute for Medical Research

P.O. Box 9653, Dar es Salaam, Tanzania

Tel.: +255 22 2121400

Mobile: +255 758 587885

Hotline: +255 22 2130770

Email: ethics@nimr.or.tz / nimrethics@gmail.com

UNIVERSITY OF GOTHENBURG

Fomu ya Ridhaa

Malengo ya utafiti na taratibu zake, madhara na faida zimeelezwa kwangu. Nimeruhusiwa kuuliza maswali na maswali yangu yote yamejibiwa nimeridhika. Nimeambiwa naweza kuwasiliana na wasimamizi wa maadili ya tafiti KCMC kama nina maswali kuhusu haki yangu kama mshiriki wa utafiti.

Ninawahakikishia kuwa nimesoma ama kusomewa taarifa ya mshiriki. Ninaelewa kuwa ushiriki wangu ni wa hiari, na kuwa ninaweza kujitoa muda wowote ule bila haki zangu stahiki kudhuriwa.
Nimefahamishwa kuwa nitapatiwa nakala ya fomu hii ya uhiari wa kushiriki kama kumbukumbu yangu. Ninakubali kushiriki utafiti huu

Jina la Mshiriki…………………………………….. Sahihi …………………….

Tarehe………………………….

Jina la mtafiti anaedodosa…………...………. Sahihi ……………….......

tarehe………………………….

Shahidi (kama yupo)………………………………..Sahihi……………………..

Tarehe……………………………
